# Supplementary material for: Single-cell and spatial transcriptomics reveal post-translational modifications in osteosarcoma progression and tumor microenvironment
Source: PLoS One. 2025 Oct 9;20(10):e0333809. doi: 10.1371/journal.pone.0333809 (PMC12510563; doi:10.1371/journal.pone.0333809)
Supplement: S1 File — S1 Table. Post-translational modifications-related genes list. S2 Table: Sequences for siRNA. S3 Table: RT-qPCR primer sequences. S1 Fig. Gene set enrichment analysis (GSEA) based on dysregulated genes of the osteoblastic cells. (A, B) GSEA analysis of dysregulated genes in osteoblastic cells based on PTMshighos and PTMslowos. S2 Fig. Intercellular communication analyses. (A) Intercellular communication pathways among PTMs-associated osteoblastic cells. (B) Correlation circle plots illustrating interactions between different cell types. (C) Heatmap showing cell–cell ligand-receptor correlations. (D) Sankey diagram visualizing ligand-receptor-transcription factor interactions in fibroblasts with higher PTMs expression. S3 Fig. Clinical practice value of CMDPTMS. (A, B) Comparison of CMDPTMS with 16 other published models in the TARGET-OS and GEO-OS cohorts. S4 Fig. Prognostic significance of CMDPTMS. (A) Nomogram predicting 1-, 3-, and 5-year overall survival in OS patients. (B) Kaplan-Meier survival analysis comparing high- and low-score groups. (C-E) Decision curve analysis assessing the clinical utility of the nomogram for 1-, 3-, and 5-year survival predictions. S5 Fig. GSEA-KEGG pathway enrichment analysis in the low CMDPTMS and high CMDPTMS groups. A. GSEA-KEGG analysis of metabolic-related pathways in the low CMDPTMS and high CMDPTMS groups. (B, C). GSEA-KEGG analysis in different CMDPTMS groups. S6 Fig. The predictive value of CMDPTMS in immunotherapy response among OS patients. (A) Differences in restricted mean survival (RMS) time at 6 months and 1-year post-treatment. (B) Differences in long-term survival (LTS) at 3 months post-treatment. (C) Distribution of CMDPTMS across immunotherapy response groups. (D) Variations in TIP process activation levels. (E) Immunotherapy response predictions based on the TIDE algorithm. (F) Immunotherapy response predictions based on the subclass mapping algorithm. (G) Distribution of CMDPTMS across immunotherapy response gr [file pone.0333809.s001.zip › S3 Table.docx]

**S3 Table:** RT-qPCR primer sequences

| Gene name | Sequence (5'-3') |
| --- | --- |
| *GAPDH* | Forward: CAGGAGGCATTGCTGATGAT |
|  | Reverse: GAAGGCTGGGGCTCATTT |
| *VIM* | Forward: TCCGCACATTCGAGCAAAGA |
|  | Reverse: TGAGGGCTCCTAGCGGTTTA |
