# Supplementary material for: Single-cell and spatial transcriptomics reveal post-translational modifications in osteosarcoma progression and tumor microenvironment
Source: PLoS One. 2025 Oct 9;20(10):e0333809. doi: 10.1371/journal.pone.0333809 (PMC12510563; doi:10.1371/journal.pone.0333809)
Supplement: Attachment — Submitted filename: Response to Reviewers.docx [file pone.0333809.s002.docx]

**Rebuttal letter**

**Editor**

Dear Editor,

We are hereby submitting a revised manuscript entitled “Single-cell and spatial transcriptomics reveal post-translational modifications in osteosarcoma progression and tumor microenvironment” (Submission ID: PONE-D-25-25008) for publication. We appreciate very much for the constructive comments and suggestions from you and the reviewers. In this revised manuscript we have taken every effort to address the comments and concerns. All the changes in the text are highlighted in red. The details of our revised manuscript, as required by Editor, reviewer 2 and 5 as below.

**Journal Requirements and Editor Comments:**

***Comment 1 of Journal Requirements:*** If the reviewer comments include a recommendation to cite specific previously published works, please review and evaluate these publications to determine whether they are relevant and should be cited. There is no requirement to cite these works unless the editor has indicated otherwise.

**Response:** We sincerely appreciate your valuable comments, and we have undertaken a careful re-examination of our references. With the exception of references 24 and 28 (DOI: 10.15212/bioi-2022-0008, DOI: 10.15212/bioi-2022-0014), which were specifically requested by the reviewers during the first peer review, all other references are directly related to our research. Accordingly, we have decided to remove the above-mentioned references.

***Comment 2 of Journal Requirements:*** Please review your reference list to ensure that it is complete and correct. If you have cited papers that have been retracted, please include the rationale for doing so in the manuscript text, or remove these references and replace them with relevant current references. Any changes to the reference list should be mentioned in the rebuttal letter that accompanies your revised manuscript. If you need to cite a retracted article, indicate the article’s retracted status in the References list and also include a citation and full reference for the retraction notice.

**Response:** Thank you for your valuable comments, we have re-examined our references. With the exception of references 24 and 28, all other references can be found in the National Center for Biotechnology Information (NCBI) databases. Accordingly, we have decided to remove references 24 and 28 from the methods section of our manuscript, as they do not affect the results or integrity of our study.

***Additional Editor Comments:*** Please revise the manuscript according to the reviewers' comments.

**Response:** Thank you for your valuable comments. We have carefully revised the manuscript in accordance with the reviewers' comments. All suggestions have been fully addressed, and the corresponding changes have been incorporated into the revised version. For clarity, all modifications in the text are highlighted in red.

**Reviewer(s)' Comments:**

***Comment 1 of Reviewer 2:*** Please spell out "*VIM*" in the abstract and main manuscript, I assume this is vimentin?

**Response:** Thank you for your valuable advice. We have already changed “VIM” to “vimentin” in line 25 of the revised manuscript.

***Comment 2 of Reviewer 2:*** Abstract needs to define PTMs highos and PTMs lowos; that is defined in the manuscript, but not in the abstract.

**Response:** Thank you for your valuable comments. We have defined PTMs highos and PTMs lowos in the Abstract section of the revised manuscript.

***Comment 3 of Reviewer 2:*** Please defined what is high-CMDPTMS group in the abstract.

**Response:** Thank you for your valuable comments. We have defined high-CMDPTMS group in the Abstract section of the revised manuscript.

***Comment 4 of Reviewer 2:*** Abstract: why do the authors mention "however, seven drugs emerged as potential therapeutic options for these 34 patients."? it doesn't seem to contradict or go the opposite direction of the high-CMDPTMS group. This phrase/ part of paragraph needs to be reviewed and adjusted to be easier to understand.

**Response:** Thank you for your valuable advice. We have changed this sentence to “Although the high-CMDPTMS group (over the median risk score) was linked to poor outcomes and diminished benefit from immunotherapy, seven drugs were identified that may offer therapeutic promise for these patients.”

***Comment 5 of Reviewer 2:*** Abstract: would change the phrase "established CMDPTMS as a powerful tool for enhancing OS prognosis prediction and optimizing immunotherapy strategies." as this single study can't "establish" the tool but could change it for something like "we are introducing the CMDPTMS as a powerful tool that should be further evaluated to enhance OS prognosis prediction..." or along those lines.

**Response:** Thank you for your valuable advice. We have changed this sentence to “We introduced that CMDPTMS may serve as a powerful tool for improving OS prognosis prediction and optimizing immunotherapy strategies.”

We revised this sentence for the following reason: it is generally not appropriate to present the limitations of a study in the abstract section. Other published studies also omit research flaws in their abstracts, such as those reported in PMID: 33976213, PMID: 34975338, PMID: 40186284, PMID: 40139615, and PMID: 39948301.

Accordingly, we have rewritten the sentence in lines 39 using the subjunctive form.

***Comment 6 of Reviewer 2:*** line 51: would change "immune checkpoint molecules" for "immune checkpoint inhibitors".

**Response:** Thank you for your valuable advice. We have changed “immune checkpoint molecules” to “immune checkpoint inhibitors” in line 54 of the revised manuscript.

***Comment 7 of Reviewer 2:*** line 88 and 89: please introduce KEGG and GSEA abbreviations, respectively here. They're introduced later in the manuscript, but it should be here instead.

**Response:** Thank you for your valuable comments. We have introduced the abbreviations of KEGG and GSEA in this sentence.

***Comment 8 of Reviewer 2:*** line 94: would indicate single cell RNA sequencing, instead of single cell sequencing. And would introduce in parenthesis the abbreviation.

**Response:** Thank you for your valuable advice. We have changed “single cell sequencing” to “single cell RNA sequencing” in line 98 of the revised manuscript

***Comment 9 of Reviewer 2:*** line 107-108: spell and add in parenthesis ST.

**Response:** Thank you for your valuable advice. We have changed “ST” to “spatial transcriptomics (ST)” in line 111 of the revised manuscript

***Comment 10 of Reviewer 2:*** line 226: were the intercellular interactions particularly found in the PTMs high or low groups or both? please clarify.

**Response:** Thank you for your valuable comments. We have rewritten this sentence to resolve these issues.

***Comment 11 of Reviewer 2:*** line 232: please add a phrase that summarizes the findings of the paragraph, as it is highly technical and difficult to see the "big picture" that it's trying to convey. Also, please clarify if the authors found that the PTMs high and low groups are both found in every OS samples, or if it's rather that some OS are predominantly PTMs high and others are rather PTMs low.

**Response:** Thank you for your valuable comments. We have rewritten lines 223, 231, 233, and 235-238 to address this issue. In addition, we have modified lines 218 and 246 to further clarify that both PTMshigh and PTMslow groups are present in all OS samples.

***Comment 12 of Reviewer 2:*** line 315: what exactly is "superior prognostic results"? (e.g., no response vs. partial vs. complete response to therapy, overall survival, progression free survival) and is it in the context of bladder cancer or osteosarcoma? if in context of osteosarcoma, how was the conversion or translation done from bladder cancer to osteosarcoma?

**Response:** Thank you for your valuable advice. We have rewritten lines 323, 327 to address this issue. The IMvigor bladder cancer (IMvigor210) cohort, used as a common external validation cohort for the prognostic model, can also be found in the following articles: PMID: 37925432, PMID: 35864548, PMID: 37214439, PMID: 36160460, PMID: 35691273, PMID: 35309925, PMID: 38715811, PMID: 39391236, and PMID: 34631713.

The code to generate S6 Figure A-C is as follows:

suppressPackageStartupMessages({

library(tidyverse)

library(data.table)

library(survival)

library(survminer)

library(ggpubr)

library(patchwork)

library(viridis)

library(ComplexHeatmap)})

module.gene = read.table("module.gene.txt") %>% pull(V1)

calRS <- function(cli,data,module.gene){

selectGenes <- intersect(module.gene,row.names(data))

selectSamples <- intersect(rownames(cli),colnames(data))

data <- data[selectGenes,selectSamples]

cli <- cli[selectSamples,] %>% cbind(t(data))

fmla <- paste0("Surv(OS.time, OS)~" ,paste0(selectGenes,collapse = '+')) %>% as.formula()

cox <- coxph(fmla, data = cli)

coef <- coef(cox)

cli$RS <- coef %*% as.matrix(data) %>% as.numeric()

RS.cut <- surv_cutpoint(cli, time = "OS.time", event = "OS",variables = "RS")$cutpoint[1,1]

cli <- cli %>% mutate(binRS = ifelse(RS > RS.cut,"High","Low")) %>% select(-selectGenes)

return(cli)}

############################# IMvigor210

IMvigor210.exp=read.table("IMvigor210.exp.txt.gz",check.names = F)

IMvigor210.cli= read.table("IMvigor210.cli.txt")

IMvigor210.cli <- calRS(IMvigor210.cli,IMvigor210.exp,module.gene)

############################# S6 figA

diff.time1<-survdiff(Surv(OS.time/30,OS)~binRS, data=IMvigor210.cli[IMvigor210.cli$OS.time/30 < 6,])

pvalue.time1 <- diff.time1$pvalue

diff.time2<-survdiff(Surv(OS.time/30,OS)~binRS, data=IMvigor210.cli[IMvigor210.cli$OS.time/30 < 12,])

pvalue.time2 <-diff.time2$pvalue

sfit <- survfit(Surv(OS.time/30, OS)~binRS, data=IMvigor210.cli)

p <- ggsurvplot(sfit,data = IMvigor210.cli,

palette = 'jco',legend.title="",

legend.labs=c("High CMDPTMS","Low CMDPTMS"),xlim=c(0,12),break.x.by=2)

p<- p$plot +

geom_vline(xintercept = 6, linetype = "dashed") +

geom_vline(xintercept = 12, linetype = "dashed") +

geom_text(x=0,y=0.1,label ="Comparison of RMS at 6-month",hjust = -0.1) +

geom_text(x=6,y=0.1,label ="Comparison of RMS at 12-month",hjust = -0.1) +

geom_text(x=0,y=0,label =sprintf("p = %.2f",pvalue.time1),hjust = -1) +

geom_text(x=6,y=0,label =sprintf("p = %.2f",pvalue.time2),hjust = -1) +

labs(x="Time(months)",y="Overall survival")

ggsave("S6 figA.pdf",p,height = 6,width = 6)

############################# S6 figB

diff.time<-survdiff(Surv(OS.time/30,OS)~binRS, data=IMvigor210.cli[IMvigor210.cli$OS.time/30 > 3,])

pvalue.time <- diff.time$pvalue

pvalue.time <- ifelse(pvalue.time<0.01,"p < 0.01",round(pvalue.time,2))

sfit <- survfit(Surv(OS.time/30, OS)~binRS, data=IMvigor210.cli)

p <- ggsurvplot(sfit,data = IMvigor210.cli,

palette = 'jco',legend.title="",

legend.labs=c("High CMDPTMS","Low CMDPTMS"),

break.x.by=3)

p <- p$plot +

geom_vline(xintercept = 3, linetype = "dashed") +

geom_text(x=3,y=0.1,label ="Comparison of LTS after 3-month",hjust = -0.1) +

geom_text(x=3,y=0,label = pvalue.time,hjust = -1) +

labs(x="Time(months)",y="Overall survival")

ggsave("S6 figB.pdf",p,height = 6,width = 6)

############################# S6 figC

p <- IMvigor210.cli %>%

filter(Response!="NE") %>%

ggboxplot(x='Response', y='RS',color = "Response",palette = 'jco',

add="jitter",add.params = list(size=3)) +

stat_compare_means(label.y = max(IMvigor210.cli$RS) + 2) +

stat_compare_means(comparisons=list(c('CR','PD'),c('CR','SD'),c("CR","PR"),

c('PR','PD'),c('PR','SD'),c("PD","SD"))) +

labs(x="",y="CMDPTMS",fill='Overall Response')

ggsave(S6 figC.pdf",p,height = 6,width = 6)

***Comment 13 of Reviewer 2:***  line 362: is there a way to know if the MG-63/U-2 OS cells correspond to the CMDPTMS high or low groups? this would be important to better understand the role of sliencing vimentin.

**Response:** Thank you for your valuable advice. We could not analyze MG-63/U-2 OS cells correspond to the CMDPTMS high or low groups, because we did not run RNA sequencing for MG-63/U-2 OS cells. We changed “To further corroborate these observations, we examined VIM expression at both mRNA and protein levels using the Human Protein Atlas (HPA) database and RT-qPCR” to “To further corroborate these observations, we examined VIM expression at both mRNA and protein levels using the Human Protein Atlas (HPA) database and RT-qPCR, given that VIM exhibited peak expression during the late differentiation stages of pseudotime analysis in both scRNA-seq and ST of OS” in order to better clarify the role of vimentin silencing.

***Comment 14 of Reviewer 2:*** line 440: would change "PD-L1 blockade" for "PD-1/ PD-L1 blockade".

**Response:** Thank you for your valuable comments. We have changed “PD-L1 blockade” to “PD-1/ PD-L1 blockade” in line 453.

***Comment 15 of Reviewer 2:*** lines 443-460: please clarify what was the finding the the seven drugs; was it that the highos group are likely to be more sensitive to these agents compared to lowos group or is it that all os cells seems to be sensitive to those seven drugs based on the CMDPTMS? also I would state all seven drugs first, then go through each drug stating their clinical utility in os or other tumors, that way you give a "big picture" up front before the detailed information.

**Response:** Thank you for your valuable comments. We have rewritten the sentence in lines 457-460 as follows to resolve these issues: In light of the limited immunotherapy efficacy observed in the high-CMDPTMS population compared with the low-CMDPTMS population, we performed an extensive screening of potential therapeutic agents, including AZD8055, erlotinib, PF-4708671, dabrafenib, ribociclib, ulixertinib and ulixertinib.1.

***Comment 1 of Reviewer 5:*** All my previous concerns have been satisfactorily addressed. I recommend acceptance for publication of this paper.

**Response:** Thank you for your positive feedback and kind recommendation. We sincerely appreciate the time and effort you dedicated to reviewing our manuscript.

Again, we thank the Editors/Reviewers’ for their efforts to improve the manuscript. We hope that the revised manuscript is now suitable for publication. If you need any more information, please let us know. We are looking forward to hearing your response.

Yours sincerely,

Prof. Tao Ding
